# Development of functional organization within the sensorimotor network across the perinatal period

**DOI:** 10.1002/hbm.25785

**Published:** 2022-01-28

**Authors:** Sofia Dall'Orso, Tomoki Arichi, Sean P. Fitzgibbon, A. David Edwards, Etienne Burdet, Silvia Muceli

**Affiliations:** ^1^ Department of Electrical Engineering Chalmers University of Technology Gothenburg; ^2^ Centre for the Developing Brain School of Biomedical Engineering and Imaging Sciences, King's College London London; ^3^ Department of Bioengineering Imperial College of Science, Technology and Medicine London UK; ^4^ Paediatric Neurosciences Evelina London Children's Hospital, St. Thomas' Hospital London UK; ^5^ Medical Research Council Centre for Neurodevelopmental Disorders King's College London London UK; ^6^ Wellcome Centre for Integrative Neuroimaging FMRIB, Nuffield Department of Clinical Neurosciences, University of Oxford Oxford UK

**Keywords:** functional connectivity, infants, neurodevelopment, prematurity, resting‐state fMRI, sensorimotor cortex, somatotopic organization

## Abstract

In the mature human brain, the neural processing related to different body parts is reflected in patterns of functional connectivity, which is strongest between functional homologs in opposite cortical hemispheres. To understand how this organization is first established, we investigated functional connectivity between limb regions in the sensorimotor cortex in 400 preterm and term infants aged across the equivalent period to the third trimester of gestation (32–45 weeks postmenstrual age). Masks were obtained from empirically derived functional responses in neonates from an independent data set. We demonstrate the early presence of a crude but spatially organized functional connectivity, that rapidly matures across the preterm period to achieve an adult‐like configuration by the normal time of birth. Specifically, connectivity was strongest between homolog regions, followed by connectivity between adjacent regions (different limbs but same hemisphere) already in the preterm brain, and increased with age. These changes were specific to the sensorimotor network. Crucially, these trajectories were strongly dependent on age more than age of birth. This demonstrates that during the perinatal period the sensorimotor cortex undergoes preprogrammed changes determining the functional movement organization that are not altered by preterm birth in absence of brain injury.

## Graphical abstract

To study the typical trajectory of development within the human brain's sensorimotor organization, we characterized maturational changes in connectivity between body region representations in a unique large dataset of high‐quality resting‐state functional MRI from 400 neonates aged across the equivalent period to the third trimester of gestation. Our results demonstrate the early presence of a crude functional organization within the sensorimotor network, that rapidly matures to achieve an adult‐like configuration by the normal time of birth. Through comparison between preterm and term born infants, we further demonstrate that this maturation trajectory is strongly driven by preprogrammed mechanisms and is not significantly altered by environmental factors.

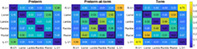

## 1 INTRODUCTION

It is increasingly appreciated that complex voluntary motor behavior is encoded in the human brain through a highly organized network of neural connectivity. A key component of this network is the sensorimotor cortex (primary somatosensory and motor cortices), which is organized in a somatotopic fashion such that the processing relating to different body parts are mapped to specific locations within the cortex (Penfield & Boldrey, 1937). Connectivity patterns between these cortical territories reflect motor experiences and are stronger between those territories related to body parts that usually cooperate during everyday functions (Ejaz, Hamada, & Diedrichsen, 2015). Specifically, homolog areas are the most connected (van den Heuvel & Pol, 2010; Stark et al., 2008) and neighboring cortical areas are also strongly connected (Long, Goltz, Margulies, Nierhaus, & Villringer, 2014; Thomas Yeo et al., 2011). This topographical specialization constitutes the backbone underlying motor skills both ontogenetically and phylogenetically (Kuehn et al., 2017).

While it is known that a crude somatotopy is already present in the sensorimotor cortex before the normal time of birth (Dall'Orso et al., 2018), it is likely that the aforementioned specific patterns of connectivity must also arise in early life to provide a substrate for the rapid development of essential motor skills in early childhood. However, detailed spatial and temporal knowledge about the early evolution of functional organization within the human sensorimotor network is currently missing, mainly because of the challenges inherent to studying the brain across the perinatal period. This limitation has been addressed through recent advances in functional magnetic resonance imaging (fMRI) methods via the Developing Human Connectome Project [dHCP: http://www.developingconnectome.org (Fitzgibbon et al., 2020)] which allow precise characterization of these maturational process across a large population of infants in the period corresponding to the third trimester of human gestation. Studying preterm infants in this cohort also provides an opportunity to directly test the potentially contrasting developmental roles of intrinsic genetic factors and environmental influences. Furthermore, understanding typical sensorimotor network development will help provide mechanistic insight into how early deviations may result in specific patterns of adverse motor outcome later in life and enable early identification of affected children when this development is atypical. Although it has been shown that disruption of neonatal functional connectivity in children with perinatal brain injury predicts later developmental outcome more reliably than structural MRI (Linke et al., 2018), current functional connectivity measures are coarse and lack specificity. A detailed analysis of the spatial organization of functional connectivity in the immature sensorimotor cortex is missing.

To study functional connectivity relationships between emerging brain representations of different body parts in early human life, we analyzed high quality resting‐state fMRI data from a large population (*n* = 400) of preterm and term infants. Functional connectivity was analyzed between four brain regions within the sensorimotor network corresponding to the left and right ankle and wrist, and a control area (the left and right visual cortex). To accurately identify the spatial organization of functional connectivity in the primary sensorimotor cortex in the neonatal brain, the limb regions were identified from functional brain responses to passive movement of the wrists and ankles in an independent group of preterm neonates (Dall'Orso et al., 2018) rather than from anatomical landmarks. We hypothesized that across the perinatal period, patterns of functional connectivity specific to the sensorimotor network would gradually mature towards the canonical adult configuration, characterized by maximal connectivity between regions of the sensorimotor network corresponding to homolog body parts in opposite hemispheres (van den Heuvel & Pol, 2010) and ipsilateral neighboring cortical areas (Long et al., 2014; Thomas Yeo et al., 2011). Our study used unprecedent temporal (from 32 to 45 weeks PMA) and spatial resolution (between cortical brain regions of the limbs), which has allowed us, to the best of our knowledge to perform the first characterization of the typical trajectory of these maturational changes in functional connectivity within the primary sensorimotor network during this critical period of early life. To provide further insight into the potential roles of nature versus nurture in sensorimotor network maturation, we also compared infants born prematurely at term equivalent age with those delivered at full term. If connectivity patterns are mediated by environmental influences, maturation would be faster in preterm born infants compared with their term born peers due to their greater exposure to environmental stimulation.

## 2 MATERIALS AND METHODS

### 2.1 Subjects

**FIGURE 1:**
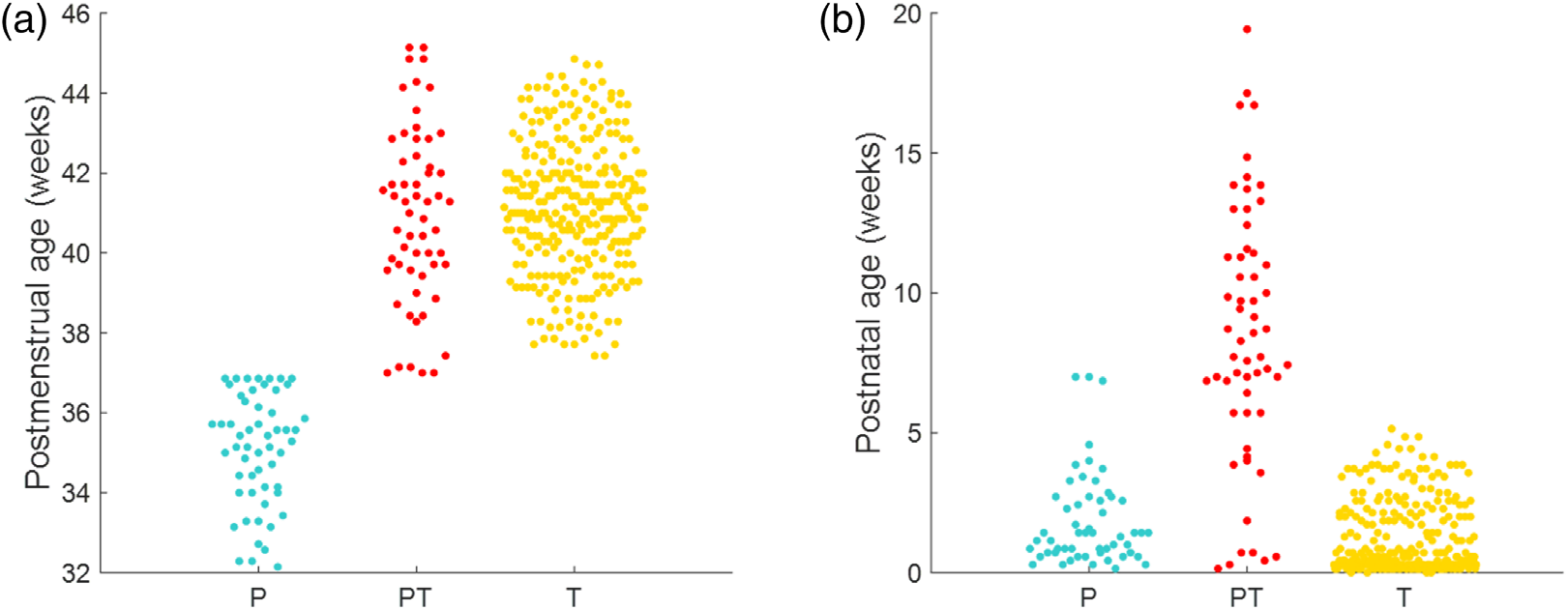
Demographic distribution of the studied infant groups: preterm (light‐blue), preterm‐at‐term (red), and term (yellow). Each dot represents a subject postmenstrual age at scan (a) or postnatal age at scan (b)

Study participants and their associated blood oxygen level dependent (BOLD) contrast functional MR images were selected from an open‐source database made available by the dHCP (2nd data release, www.developingconnectome.org), a large Open Science program approved by the UK National Research Ethics Authority (14/LO/1169). Informed written consent was obtained from all participating families. Infants were studied during natural sleep (nonsedated) following feeding. From the large cohort available, infants who had incidental findings with possible or likely clinical significance (such as intracranial hemorrhage or congenital malformations), and those whose data quality did not meet the criteria explained in the *functional MRI data preprocessing* subsection were excluded. In this study, a total of 377 healthy infants were included with median gestational age (GA) at birth 39.6 weeks (range: 23 + 0 to 42 + 2 weeks + days; 212 male). Twenty‐three infants were included twice as they were studied both at preterm and term equivalent age, yielding a dataset of 400 images with postmenstrual age (PMA) at scan ranging from 32 + 1 to 45 + 1 weeks + days (median 40.9 weeks; 225 male). Of this group, 54 infants were born and studied in the preterm period [PMA (median [range]): (35.4 [32 + 1 to 36 + 6] weeks + days)], 58 infants were born preterm but were studied at term equivalent age [PMA (median [range]): (41.1 [37 + 0 to 45 + 1] weeks + days)], and 288 were infants born at full term [PMA (median [range]: (41.1 [37 + 3 to 44 + 6] weeks + days)]. Demographic distribution of the studied infant groups is shown in Figure 1, and specific details of each subject are reported in the Supporting Information.

### 2.2 MRI data acquisition

Data acquisition took place at the Evelina Newborn Imaging Centre at St. Thomas' Hospital, London, UK, using a 3‐T Philips Achieva system (Philips Medical Systems, Best, The Netherlands) and a dedicated 32‐channel neonatal head coil and positioning system (Hughes et al., 2017). Subject preparation was optimized to ensure safety and comfort of neonates enabling high imaging quality and success rate (Hughes et al., 2017). Fifteen minutes of high temporal resolution resting‐state BOLD fMRI were obtained using a gradient‐echo‐planar imaging (EPI) sequence with multiband excitation (TE = 38 ms; TR = 392 ms; MB factor = 9; 2.15 mm isotropic voxel resolution; total 2,300 volumes; Price et al., 2015). High‐resolution (0.8 mm isotropic) T1‐weighted and T2‐weighted anatomical images were also acquired in the same scan session and were used for registration purpose (Fitzgibbon et al., 2020).

### 2.3 Functional MRI data preprocessing

fMRI data were preprocessed using a specific pipeline developed for the dHCP described in more detail in Fitzgibbon et al. (2020). This included correction for susceptibility distortion, volume and slice misalignment, spin‐history, and multiband artifacts with field inhomogeneity correction (FSL TOPUP; Andersson, Skare, & Ashburner, 2003), slice‐to‐volume and rigid‐body registration (FSL EDDY; Andersson et al., 2017), nuisance regression (24 head motion parameters), high‐pass filtering (cut‐off period of 150 s) and FIX denoising (FMRIB's ICA‐based X‐noiseifier; Salimi‐Khorshidi et al., 2014). The latter was used to remove head‐movement, multiband artifact, sagittal sinus, artery, and CSF pulsation, as well as other unclassified noise. The filtered and denoised 4D functional images were registered to the subject's native T2‐weighted space (using boundary‐based registration) and then nonlinearly (using a diffeomorphic T1w/Tw estimation) registered to a standard space (40‐week template from the dHCP volumetric atlas; Schuh et al., 2018) accounting for extra considerations needed for the newborn brain (Fitzgibbon et al., 2020). Ninety out of five hundred and ninety subjects were excluded due to more than 10% of the acquired volumes being identified by the dHCP pipeline as motion outliers when the root mean square difference between successive volumes was higher than 1.5 IQR above the 75th percentile (Fitzgibbon et al., 2020). An additional quality check of the signal intensity within the sensorimotor and visual cortices was performed by comparing the BOLD value of each voxel of interest to the mean BOLD value of the cerebrospinal fluid (CSF; Westbrook, Roth, & Talbot, 2005). Finally, the number of voxels that suffered from signal dropout (voxel's BOLD < CSF's BOLD) was counted and only those subjects who had at least 80% of voxels within the expected range for a biological response in both the sensorimotor network and primary visual cortex (V1) were included (*n* = 447). Fifty three out of five hundred subjects were excluded as greater than 20% of voxels were affected by poor signal in either the sensorimotor or V1 region due to signal dropout particularly that caused by the confluence of venous sinuses in the inferior occipital and superior–anterior cerebellum (Fitzgibbon et al., 2020). Forty seven images were excluded due to incidental findings with possible or likely clinical significance and/or sedation during the data acquisition.

### 2.4 Region of interest selection

This study investigated functional connectivity among brain regions corresponding to bilateral limbs (left and right wrist and ankle, in pairs), and between each of these four regions and the sensorimotor network in the opposite hemisphere. Two control regions in V1 were considered for comparison. Therefore, the analysis made use of several regions of interest (ROIs) that were selected from independent published studies on neonates and registered into a standard space common to fMRI data. The ROIs corresponding to the sensorimotor network and V1 were identified from the resting‐state analysis of a large group of infants studied within the dHCP (Eyre et al., 2021). Reference network masks were taken from those defined from the oldest infants scanned at 43.5–44.5 weeks PMA as previously described (Eyre et al., 2021). These masks were also suitable for our study, as the networks would have the most mature patterns of connectivity and thus avoided the possibility of underestimating connectivity in the oldest infants if the masks had been derived from younger infants or averaged across all ages. The sensorimotor network encompassed the somatosensory, medial motor, and motor association networks, which were combined and then split into the two hemispheres excluding the midline voxels and any those outside the brain. Similarly, the V1 network was divided between left and right hemisphere. The limb ROIs were identified in our previous work that measured the functional response to passive movement of wrists and ankles in a group of preterm neonates (Dall'Orso et al., 2018) and made publicly available at www.brain-development.org/somatotopicmap/. Those masks were registered from their original 34‐week template to the 40‐week template used in this study. The final ROIs did not include: (a) any overlapping voxel between each other to avoid possible confounds (they originally shared some voxels between adjacent areas in the cortex such as right wrist and ankle), and (b) voxels that fell outside the sensorimotor network mask described above to avoid the inclusion of signal coming from unrelated areas.

### 2.5 Seed‐based connectivity analysis between limbs and contralateral sensorimotor network

**FIGURE 2:**
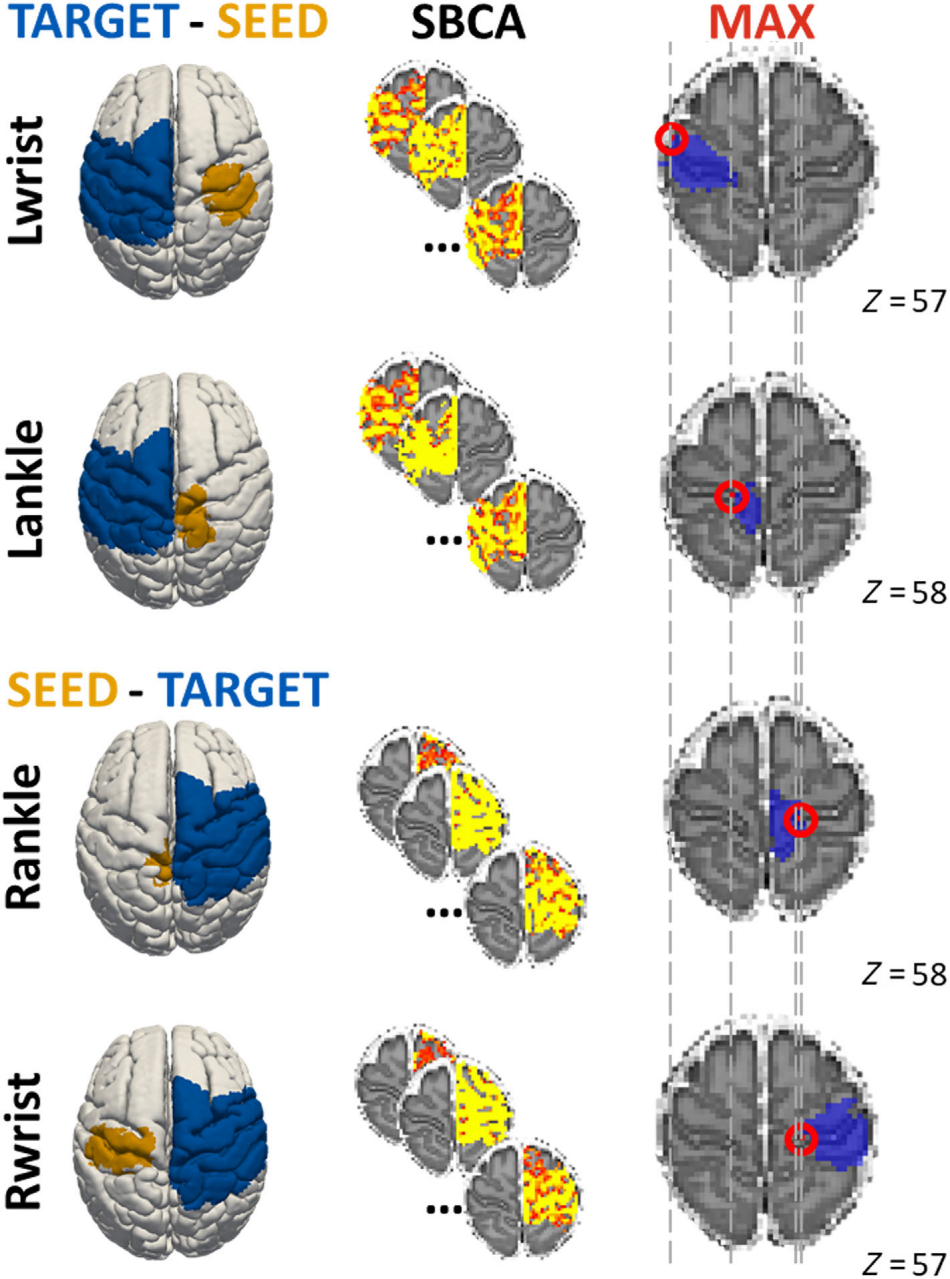
Results of the seed‐based connectivity analysis (SBCA). The different steps of the analysis are represented from left to right. Each region of interest of body parts (first column, ochre: left wrist, left ankle, right ankle, and right wrist) served as the seed region and the contralateral sensorimotor network (blue) as the target region. A temporal correlation analysis between the voxels of the seed and target regions was performed for each subject and *z*‐transformed (second column). A group analysis (*n* = 400) was performed for each seed to identify the voxel of maximal connectivity (third column, red). The homolog mask of the seed (blue) is represented together with the maximal connectivity voxel (red) to show their proximity. Brain images are shown in the anatomical view

To test whether the strongest functional connections in the neonatal sensorimotor network were between homolog regions in opposite hemispheres, inter‐regional connectivity between individual body part representations and the contralateral resting‐state sensorimotor network was examined. Specifically, a seed‐based connectivity analysis between brain regions corresponding to the left/right wrist and ankle (localized in the right/left hemisphere) and the contralateral left/right sensorimotor network was performed. The location of maximal connectivity between each of the four limb ROIs and the contralateral sensorimotor network by means of seed‐based connectivity analysis was found using tools implemented in the FMRIB's Software Library (FSL, www.fmrib.ox.ac.uk/fsl) (Smith et al., 2004). Specifically, the FSL's built in function (*fsl_sbca*) was used to estimate connectivity between each limb ROI (seed) and the contralateral sensorimotor network (target region; Figure 2). Correlation maps of each subject and seed were then converted to normal distribution by means of the Fisher's *z*‐transform. A voxel‐wise group analysis of the *z*‐transformed correlation maps was performed using a one‐sample *t*‐test by means of the permutation methods as implemented in FSL Randomize v2.1 (5,000 permutations) (Winkler, Ridgway, Webster, Smith, & Nichols, 2014) and using threshold‐free cluster enhancement (Smith & Nichols, 2009). Additional covariates (including GA, PMA, sex, and number of motion outliers) were added to the model. The location of maximal connectivity was then identified in voxel coordinates from the resulting significant group connectivity map.

### 2.6 Connectivity development across the perinatal period

A second more detailed connectivity analysis was performed to investigate specific age‐related patterns of development. The mean timeseries of resting state fluctuations were extracted from the six different ROIs corresponding to the four body parts (left/right wrist/ankle) and the two control areas (left/right V1). Partial correlation was used to estimate functional connectivity between two ROIs, as it removes mutual dependencies on common influences from other brain ROIs (Marrelec et al., 2006). A partial correlation matrix was computed between all pairs of ROIs for each subject and then converted to a *z*‐statistic using the Fisher's *z*‐transform. To test the effect of PMA on the correlation strength between each pair of regions (apart from the left and right V1), a linear regression was used while accounting for the possible effect of postnatal age, the interaction term of those two predictors, and the amount of head motion. The level of statistical significance was set at *α* = 0.01 and *p*‐values were corrected for multiple comparisons (Bonferroni correction, *p*‐value × 14 < .01). Further, subjects were divided into age groups spanning 2–4 weeks PMA (32 ≤ PMA < 34, *n* = 11; 34 ≤ PMA < 36, *n* = 27; 36 ≤ PMA < 38, *n* = 31; 38 ≤ PMA < 40, *n* = 71; 40 ≤ PMA < 42, *n* = 153; 42 ≤ PMA < 46, *n* = 107) for a qualitative evaluation of the evolution of the inter‐regional connectivity across the whole perinatal period.

### 2.7 Inter‐regional connectivity analysis by age groups

Preterm birth (delivery before the 37th week of gestation) is associated with an increased risk of adverse neurodevelopmental outcome and, in some cases, long‐lasting alterations in functional and structural connectivity (Batalle et al., 2019; Eyre et al., 2021). Subjects were split in three main groups for a quantitative assessment of preterm birth in terms of the statistical difference in connectivity between ROIs: preterm (*n* = 54), preterm born studied at term equivalent age (*n* = 58) and term born (*n* = 288) infants. An average partial correlation matrix was computed for each age group for visualization purposes. The partial correlation coefficients of the preterm, preterm‐at‐term, and term age groups were pooled into four categories reflecting the cortical reciprocal locations: Homologs (e.g., left/right ankle), Adjacents (ipsilateral wrist/ankle), Distals (e.g., left wrist/right ankle), and V1‐controls (any body part and V1). The functional connectivity patterns of the term brain were characterized by comparison of the connectivity strength between the four categories using a rank‐based nonparametric test (Kruskal–Wallis) and a post hoc comparisons using the Tukey HSD test. Further, Kruskal–Wallis tests and post hoc comparisons using the Tukey HSD tests were carried out to assess the effects of prematurity on the connectivity strength in each cortical location at preterm and term equivalent age time points. The 23 subjects with repeated measures were excluded from this analysis, while were analyzed separately using a paired *t*‐test which provided longitudinal information between the preterm and term time. We chose the level of significance to be consistent with the other statistical analysis (*α* = 0.01) and corrected the *p*‐values for multiple comparisons using Bonferroni correction. The analysis described in the last two sections was performed in MATLAB R2020a (The Mathworks Inc., Natick, Massachusetts).

## 3 RESULTS

### 3.1 Homolog regions are maximally connected

The seed‐based connectivity analysis yielded group connectivity maps for each ROI corresponding to individual limbs (detailed statistical *p*‐values are reported in Table S1). In agreement with our first hypothesis, the site of maximal connectivity from the group map spatially localized in the opposite cerebral hemisphere in a somatotopic manner. Specifically, we found that the voxel with maximal connectivity overlapped with or were within 1 voxel from the homolog ROI for all of the limb representations (Figure 2). Furthermore, we found a significant increase of connectivity (*p* < .005, two‐tailed) with GA and PMA, while sex was not significantly related to these patterns of connectivity. Taken together, this suggests that even in the neonatal brain there are strong functional connections between homolog brain regions, and that the strength of this relationship is significantly changing with age.

### 3.2 Age‐related changes in functional connectivity

**FIGURE 3:**
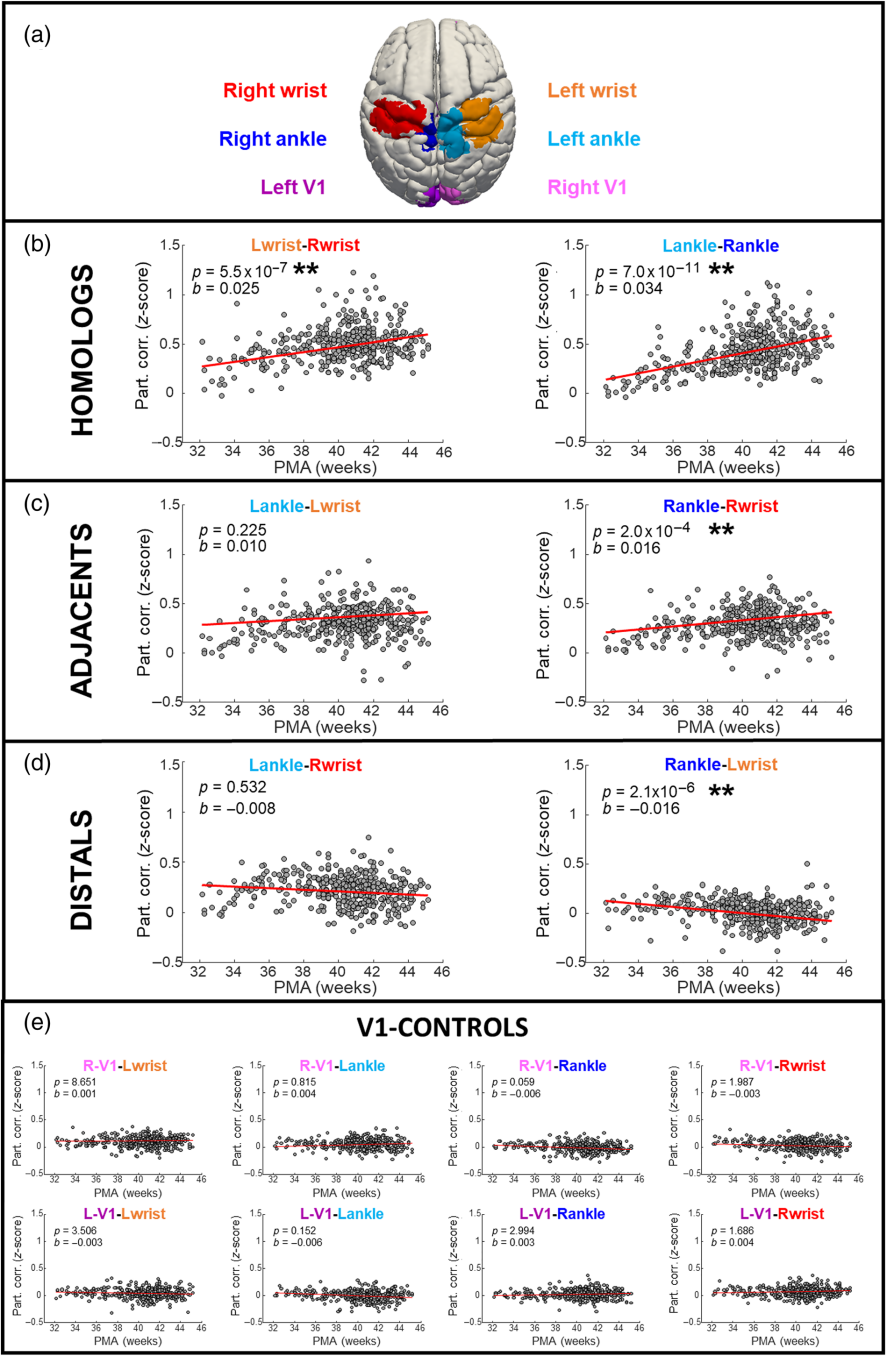
Age‐related changes in functional connectivity within the sensorimotor network. (a) Representation of regions of interest from which pairwise partial correlation was computed. (b–d) Partial correlation coefficients (*z*‐transformed) between the sensorimotor network representation of pairs of limbs plotted against postmenstrual age (PMA) and (e) between limbs and control regions. Each dot represents a subject (*n* = 400) and the red line the linear regression. Bonferroni correction was applied to *p*‐values and the two stars indicate the significance (p* 14 < .01). In keeping with our hypotheses, functional connectivity increased between homologs (same body part but different brain hemisphere); increased or remained unchanged between adjacents (different body part but same hemisphere); remained unchanged or decreased between distals (different body part and different hemisphere). In contrast to the inter‐limb connectivity, functional connectivity between limbs and control areas (left/right V1) was not affected by age in any of the cases. L, left; R, right

To assess how functional connectivity between limb ROI pairs evolves across the perinatal period, we extracted the mean timeseries from each of the four limb ROIs and two control regions (the left and right primary visual cortex; Figure 3a), and estimated functional connectivity by calculating the pairwise partial correlation between each pair of ROIs. While the regression between the *z*‐transformed partial correlation coefficients and PMA (correcting for postnatal age at study, interaction between postnatal age‐PMA, and head motion) showed no effect of postnatal age, functional connectivity strength changed significantly with PMA (p* 14 < .01 Bonferroni‐corrected) according to distinct patterns. An increase of connectivity with PMA was found between homolog regions (i.e., left/right wrist and left/right ankle; Figure 3b) followed by a milder increase between adjacent cortical areas in the left hemisphere (right wrist/ankle; Figure 3c). In contrast, functional connectivity between the left wrist and right ankle representations (distal cortical areas) decreased with increasing PMA, and connectivity between the right wrist and left ankle did not change with PMA (Figure 3d). Importantly, there was no effect of PMA on the correlation strength between any of the limbs and the control regions in the visual cortex (Figure 3e), supporting that these changes are not the result of a global connectivity increase but a specific feature of sensorimotor network development (detailed statistical *p*‐values are reported in Table S2).

To help further differentiating the evolution of these changing connectivity patterns across the sensorimotor network as a whole, the connectivity matrices of the average partial correlation coefficients across subjects were calculated split in six different age groups (32 ≤ PMA < 34; 34 ≤ PMA < 36; 36 ≤ PMA < 38; 38 ≤ PMA < 40; 40 ≤ PMA < 42; 42 ≤ PMA < 46, Figure S1). In keeping with the trends identified by the regression analysis, connectivity between homolog regions in the opposite hemisphere (elements on the right diagonal) was seen to become stronger with age. Connectivity between adjacent cortical regions (elements on the subdiagonal) was also seen to increase with age, but to a lower extent than the former. Conversely, connectivity decreased or remained unchanged between spatially distant areas. In agreement with the regression analysis results, connectivity between both visual cortices and all limb ROIs did not change during the 13‐week study period.

### 3.3 The effect of preterm birth

**FIGURE 4:**
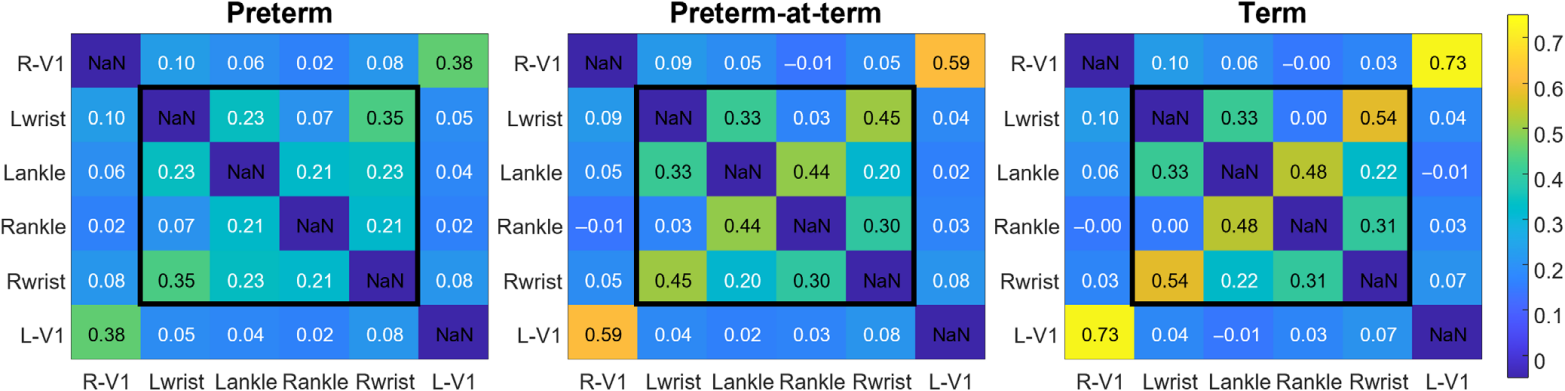
Partial correlation matrices of resting fluctuations between different cortical regions. The inner square highlights regions of the resting‐state sensorimotor network while the outer shell shows the connectivity between limbs and a control area (visual cortex, V1). The three matrices have been obtained by averaging the individual *z*‐transformed partial correlation matrices of 54 preterm, 58 preterm‐born studied at term equivalent age, and 288 term infants, respectively. L, left; R, right

To assess the effect of preterm birth at term equivalent age, subjects were clustered in three groups: infants who were born and scanned preterm (*n* = 54), infants born preterm and studied at term equivalent age (*n* = 58), and full‐term infants (*n* = 288). Importantly, infants in the preterm‐at‐term and term group had comparable PMA (Mann–Whitney *U* test: *p* = .5375) but different postnatal age at scan (Mann–Whitney *U* test: *p* = 5.8 × 10^−5^); Figure S1). Figure 4 shows the average of the individual *z*‐transformed partial correlation matrices for the three groups.

Firstly, we characterized the inter‐regional connectivity pattern of term infants and then investigated how this pattern differs in preterm‐born infants at the preterm and term‐equivalent age time‐points. We divided the correlation coefficients into four categories (Homologs, Adjacents, Distals, and V1‐controls) according to the trends previously identified by the regression analysis, and qualitatively from the correlation matrices (Figures 3 and S1). Within the term infant group, we found that connectivity strength was significantly different for each of the four categories [*H*(3) = 2,128.1, *p* < .0001], with the strongest connections among Homologs, followed by that of Adjacents, then Distals and finally V1‐controls (medians: 0.49, 0.32, 0.09, and 0.04, respectively). Once we defined this rank, we then investigated the effect of preterm, preterm‐at‐term, and term groups on each of the reciprocal cortical locations using nonparametric Kruskal–Wallis *H* tests. Twenty‐three subjects with repeated measures were excluded from this analysis and analyzed separately with paired *t*‐tests.

**FIGURE 5:**
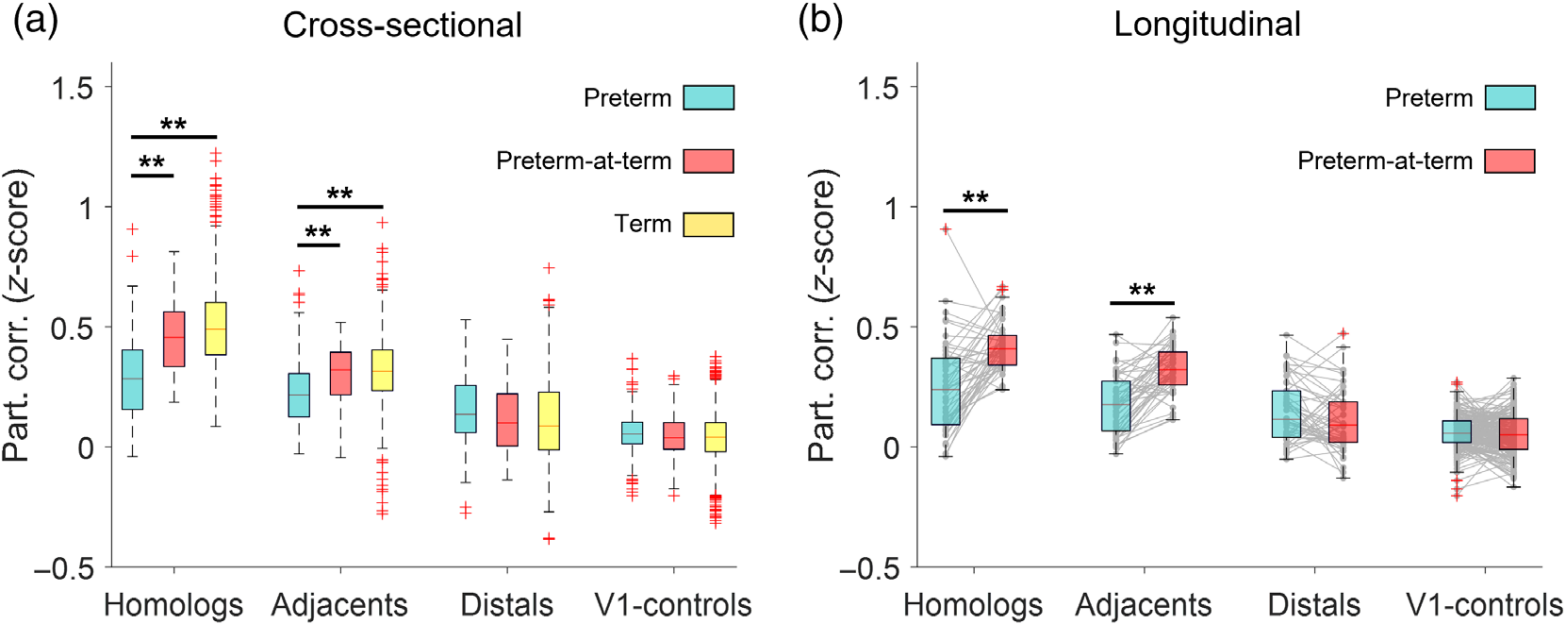
Age of birth and scan effects of functional connectivity. (a) Effect of age on the partial correlation within brain regions (Homologs, Adjacents, Distals, and V1‐controls). Significant differences (*p* < .01) are highlighted with the stars. The group of preterm infants differs from both those born preterm and scanned at term equivalent age and the term infants in the case of Homologs and Adjacents brain regions. (b) Longitudinal evolution of functional connectivity in 23 infants scanned at both the preterm and term equivalent age time points. Connectivity between Homologs and Adjacents brain regions significantly increased with age (paired *t*‐tests, p* 4 < .01)

Kruskal–Wallis tests identified significant group differences in functional connectivity between Homologs [*H*(2) = 97.33, *p*‐corrected = 2.93 × 10^−21^] and Adjacents [*H*(2) = 37.47, *p*‐corrected = 2.93 × 10^−8^]. Mean ranks of all groups and tests are reported in Table S3. The results of the post hoc comparison using the Tukey HSD test are shown grouped together in Figure 5a. Specifically, we observed a significantly lower connectivity between Homologs in preterm (median: 0.28) versus both preterm‐at‐term and term infants' groups (median: 0.46 and 0.49), and similarly significantly lower connectivity between Adjacents in preterm (median: 0.22) versus both preterm‐at‐term and term groups (medians: 0.32 and 0.32). Interestingly, the preterm‐at‐term group showed a more similar pattern of functional connectivity to the term group rather than to the preterm group. Functional connectivity between Distals was not significantly different across the three groups (medians: 0.14, 0.10, and 0.09 for preterm, preterm‐at‐term, and term groups, respectively). In agreement with the regression results (Figure 3e), connectivity between limbs and V1 control areas was similar for the three groups (medians: 0.05, 0.04, and 0.04 for preterm, preterm‐at‐term, and term, respectively).

Our cohort of neonates included 23 subjects who were scanned longitudinally (both when preterm and later at term‐equivalent age as detailed in Table S4). We investigated longitudinal changes in this small group of infants with a paired *t*‐test for each category and corrected the p‐values from multiple comparisons (Bonferroni correction). The results are depicted in Figure 5b. We observed a significant increase in connectivity in both Homologs (*p* = 2.5 × 10^−6^) and Adjacents across the two scans (*p* = 4.8 × 10^−9^). As in all other analyses, connectivity of Distals and V1‐controls did not change (*p* = .334 and *p* = .708, respectively).

## 4 DISCUSSION

The resting‐state sensorimotor network is often considered to be a single coherent network, however, it is suborganized into specific patterns related to somatotopy (Long et al., 2014). Our results illustrate that in the neonatal brain (as in the mature brain), this configuration is already present and rapidly matures across the perinatal period. We further found that across this juncture, functional connectivity between homolog territories in opposite hemispheres rapidly increases with age, in keeping with known increases in long‐range connectivity (van den Heuvel et al., 2015; Keunen, Counsell, & Benders, 2017). Short‐range connectivity between sensorimotor network regions related to adjacent body parts was also seen to increase with age, although to a lesser extent. On the other hand, connectivity between spatially disparate areas in the brain (i.e., hand and foot representations related to opposite sides of the body) decreased or remained unchanged with increasing age. Notably, connectivity between sensorimotor network regions and control areas (V1) was low and unchanged across the entire study period indicating that the observed age‐related changes in connectivity were specific to the sensorimotor network. This specific pattern of organization became even more evident with the transition from the preterm to term periods, highlighting the key importance of this stage for the establishment of the sensorimotor network's life‐long organization. Importantly, we found that at term equivalent age, preterm‐born infants had acquired a similar organization as infants born at full term, suggesting that the development of sensorimotor network connectivity patterns is largely predetermined and guided by intrinsic processes, rather than directed by external influences.

### 4.1 Interhemispheric functional connectivity

In this study, we found strong functional connectivity that increases with age between homolog regions in the right and left hemispheres (Figures 3, 4, 5). Across the lifespan, these interhemispheric connections between sensorimotor regions are necessary to enable functional integration, bilateral motor coordination and facilitate learning (Doron & Gazzaniga, 2008; McGregor, Cashaback, & Gribble, 2016; Wahl et al., 2007). Although somatotopic responses can be readily identified in the preterm brain (Dall'Orso et al., 2018), more complex and bilateral patterns of response are established across the perinatal period (Allievi et al., 2016) in line with known maturational changes in the interhemispheric white matter connections of the corpus callosum (Broekman et al., 2014; Dubois et al., 2014). This is similarly reflected in the sensorimotor resting state network which initially appears unilateral in very preterm infants but becomes symmetrical by term equivalent age (Doria et al., 2010; Eyre et al., 2021; Fransson et al., 2007). Of interest, transcallosal connections are not the sole source of interhemispheric structural connectivity, as individuals without an intact corpus callosum (either because of congenital agenesis or surgical callosotomy) surprisingly have preserved patterns of interhemispheric functional connectivity, possibly via thalamic influences (Johnston et al., 2008; Mancuso, Uddin, Nani, Costa, & Cauda, 2019; Roland et al., 2017). The thalamus is known to play a significant role in network communication (Bell & Shine, 2016), and ascending coordinated thalamocortical activity can stimulate interhemispheric cortical coherence (Johnston et al., 2008). Of relevance to our study period, thalamocortical projections are rapidly developing across the last trimester of human gestation (Kostović & Jovanov‐Milošević, 2006), and as thalamo‐motor connectivity increases, interhemispheric resting‐state sensorimotor network connectivity also strengthens (Doria et al., 2010; Thomason et al., 2015). Together, this maturing pattern of interhemispheric connectivity likely enables efficient integration of ascending sensory information in early human infancy, and expedites motor learning by transferring new skills acquired from one side of the body to the other (Hinder, Carroll, & Summers, 2013).

### 4.2 Rapid changes in functional connectivity in sensorimotor regions

Recent studies have consistently demonstrated that primary functional networks and the associated structural architecture seen in the adult brain can be identified in the neonatal brain, albeit in immature form (Cao, Huang, & He, 2017; Fransson, Åden, Blennow, & Lagercrantz, 2011; van den Heuvel et al., 2015). Trends of maturation, characterized by increases in integration and decreases in segregation, are observed through infancy and into childhood, starting first within systems which relate to the processing related to primary sensory and motor functions, and later progressing to those though to subserve higher order functions (Cao et al., 2017; Fair et al., 2009; Huang et al., 2015). Consistent with this, while primary sensory and motor networks appear immature during the preterm period (Doria et al., 2010; Smyser et al., 2010; Thomason et al., 2015), they have an adult‐like topology by the normal time of birth (Doria et al., 2010; Fransson et al., 2007; Fransson et al., 2011). We find that this process not only encompasses increases in long‐range connectivity (from the preterm to term equivalent time‐point) but further that connectivity patterns evolve in a nonuniform manner. To our knowledge, our study is the first to characterize the typical trajectory of these developmental changes within the sensorimotor network maturation at a fine‐grained temporal (from 32 to 45 weeks PMA) and spatial resolution (between cortical brain regions of the limbs). Although we observed an inverse relationship between age and connectivity strength among distal cortical areas (Figure 3d), a group effect (preterm, preterm‐at‐term, and term) was not found (Figure 5) possibly due to the “nonspecificity” of combining all distal pairs of regions into a single measure. Specifically, unbalanced maturational trends in connectivity between the two pairs of distal cortical regions were seen, with connectivity between the right ankle and left wrist significantly decreasing with age, while connectivity between left ankle and right wrist remained unchanged (see Figure 3d). Similarly, we also observed an asymmetrical pattern, with increases in connectivity between adjacent cortical regions reaching significance in the left hemisphere but not within the right hemisphere (Figure 3c). These results are in agreement with known asymmetry in resting‐state networks, including the sensorimotor network, with the level of lateralization shown to be age dependent (Agcaoglu, Miller, Mayer, Hugdahl, & Calhoun, 2015). We thus suggest that future work could further investigate the laterality in the sensorimotor developing cortex and how this relates to later motor behavior.

### 4.3 Effects of preterm birth on maturation of connectivity by term equivalent age

Although the resting‐state sensorimotor network can be readily identified in human neonates (Doria et al., 2010; Fransson et al., 2007; Fransson et al., 2011; Smyser et al., 2010; Thomason et al., 2015), it is still unclear whether the establishment of the underlying sensorimotor connectivity is mediated by experience or follows a programmed trajectory. In support of the former, one might expect higher patterns of connectivity in preterm infants at term equivalent age due to greater ex utero experience and environmental stimulation. However, the preterm‐born infants in our study exhibited strong and highly organized patterns of connectivity at term equivalent age which crucially was not significantly different to those delivered at full term (Figure 5a). Significant maturation was also confirmed in a smaller group of infants scanned longitudinally at both the preterm and term equivalent age time points (Figure 5b). These results suggest that in the absence of brain injury, the trajectory of development is strongly coupled with age and proceeds in a highly programmed manner even following preterm birth. This is also supported by behavioral studies looking at patterns of general movements which were only minimally altered by postnatal experience (Hadders‐Algra, 2004; Hadders‐Algra, 2018). Therefore, although it is clear that the acquisition of specific motor skills relies on environmental influences postnatally and through childhood (Thelen, 1994), our results suggest that the major cortical architecture might already be in place before the time of birth to enable rapid learning and the acquisition of new skills during later infancy. Accordingly, our results show that some degree of interconnection between somatotopic areas is already present in preterm infants (see correlation between Homologs and Adjacents in Figures 3 and 4). The initial formation of this somatosensory map is thought to be guided by sensory‐driven neuronal activity which begins in utero triggered by spontaneous movements (Khazipov & Milh, 2018) with isolated arm movements already visible at 10 weeks of gestation (de Vries, Visser, & Prechtl, 1982) and a full repertoire of body movements seen at about 16 weeks (Lüchinger, Hadders‐Algra, van Kan, & de Vries, 2008). Touch receptors start to form in the palms of the hands by week 10–10.5, the soles of the feet by week 10.5–11, upper arm and forearm by week 11, thighs and legs by week 11–12 (Humphrey, 1978). While initial fetal movements are simple and isolated, during the third trimester, after thalamocortical afferent reach their target in the cortical plate and the subplate regresses, movements become writhing and increasingly more complex and fluid (Hadders‐Algra, 2018). Taken together, this suggests that the anatomical substrate underlying transmission of somatosensory input to the cortex would have already been established in the preterm subjects included in our study and may have contributed to the formation of a primitive somatotopy that is further refined in the subsequent months (Figures 4 and 5). While in a previous study of resting‐state networks preterm born infants exhibited the same network characteristics as their term born peers when reaching the same age (Doria et al., 2010), other studies have found that preterm infants have more immature patterns of network topography and reduced thalamocortical connectivity in comparison to their term born peers (Eyre et al., 2021; Smyser et al., 2010; Toulmin et al., 2015). This suggests that there are distinct trajectories of development that occur at different times in the maturing brain, and that they might be specifically affected by prematurity in different ways.

### 4.4 Study relevance and future directions

There is growing evidence that changes in functional connectivity strength, network density, global and local efficiency, and topology reflect brain maturation and that altered network organization is associated with adverse neurodevelopment (Arichi et al., 2014; Batalle et al., 2016; Eyre et al., 2021). In infants with hemorrhagic parenchymal infarction for example, interhemispheric functional connectivity at term equivalent age was largely preserved in those with good developmental outcome but disrupted in those who later developed cerebral palsy (Arichi et al., 2014). Disruption of neonatal functional connectivity in children with perinatal brain injury has also been shown to be an even more reliable predictor of later developmental outcome than structural MRI appearance and/or other clinical measures (Linke et al., 2018). Although characterizing rs‐fMRI connectivity measures has the potential to predict later motor behavior, current functional connectivity measures are only crude indicators which correlate with motor outcome and lack specificity. In this context, detailed analysis of the spatial organization of functional connectivity such as that described in our study may provide more specific information about the integrity of the sensorimotor system and consequently more precisely predict behavioral outcome. For example, abnormal topography of functional activity has been reported at the level of individual finger representations in subjects with dystonic posture of the hand, with the level of disruption correlating with the degree of neurological difficulties (Bara‐Jimenez, Catalan, Hallett, & Gerloff, 1998). Moreover, differences in intrahemispheric or interhemispheric functional connectivity have also been suggested to potentially explain differences in more subtle symptomatology such as ipsilateral or contralateral coordination deficits (Volman, Laroy, & Jongmans, 2006). Here we provide a benchmark value of early patterns of functional connectivity, with which a crucial next step will be to investigate how alterations in these patterns correlate with specific neurodevelopmental sequelae to confirm its potential diagnostic and prognostic value.

### 4.5 Limitations

Additionally, the selected limb ROIs were generated in a 34‐week template as they were derived from a previous study with an independent cohort of preterm infants (Dall'Orso et al., 2018) which were then registered to the 40‐week template for the purpose of this study. In contrast, the sensorimotor and V1 ROIs where derived directly from the 40‐week brain (Eyre et al., 2021). Although ideally we would have liked to have consistent sources for these ROIs, the limb representation in younger infants is likely to also be representative of older infants as the size of the main cluster of sensorimotor response increases from the preterm to the term‐equivalent period (Allievi et al., 2016).

In this study we grouped the connectivity measures into four categories. While those have been chosen according to our hypotheses, it is inevitable that there might be differences within the categories themselves. As it can be observed in the regression analysis (Figure 3), there were asymmetries when looking at specific trends in both Adjacents and Distals, and future studies should investigate those differences in more detail.

We acknowledge that there is unavoidable clinical heterogeneity in preterm infants that, if not accounted for, can confound normal and abnormal brain function. In our study, we have tried to reduce this potential source of unexplained variance by only studying preterm infants without clear patterns of brain injury or a history of critical illness. Having a cohort of as healthy as possible preterm infants enabled us to hypothesize that any difference between the preterm‐at‐term and term groups was largely driven by postnatal exposure. However, it is clearly challenging to disentangle potential negative effects associated with preterm birth from other effects associated with normal sensory exposure, making it an inherent limitation of the study.

## 5 CONCLUSIONS

In conclusion, we have identified a clear maturational change in functional connectivity within the sensorimotor network across the perinatal period culminating in a specific, adult‐like organization by the normal time of birth. This highlights the importance of this period for the establishment of the sensorimotor network with clear implications for motor behavior into childhood. Interestingly, by term equivalent age, we found that healthy preterm infants had similar patterns of connectivity to those delivered at full term, suggesting that the establishment of this organization is highly programmed in the human brain and relatively undisturbed by external environmental input.

## CONFLICT OF INTEREST

The authors declare no conflicts of interest.

## AUTHOR CONTRIBUTION

**Sofia Dall'Orso:** Conception, design and analysis of data, interpretation of data, writing—first draft and figures, writing—review and editing. **Tomoki Arichi:** Conception, design and analysis of data, interpretation of data, writing—review and editing. **Sean P. Fitzgibbon:** Interpretation of data, writing—review and editing. **A. David Edwards:** Interpretation of data, writing—review and editing. **Etienne Burdet:** Interpretation of data, writing—review and editing. **Silvia Muceli:** Conception, design and analysis of data, Interpretation of data, writing—review and editing.

## Supporting information


**Appendix S1** Supporting Information.

**Appendix S2** Supporting Information.

**Figure S1** Partial correlation matrices of resting fluctuations between different cortical regions. The inner square highlights regions of the resting‐state sensorimotor network while the outer shell shows the connectivity between limbs and a control area (visual cortex). Matrices have been obtained averaging the individual *z*‐transformed partial correlation matrices grouped in different age groups (32 ≤ PMA < 34, *n* = 11; 34 ≤ PMA < 36, *n* = 28; 36 ≤ PMA < 38, *n* = 31; 38 ≤ PMA < 40, *n* = 71; 40 ≤ PMA < 42, *n* = 152; 42 ≤ PMA < 46, *n* = 107).

## Data Availability

All data needed to evaluate the conclusions in the article are present in the article and/or the Supporting information. FMRI data are available to download from www.developingconnectome.org.
