# Supplementary material for: Development of functional organization within the sensorimotor network across the perinatal period
Source: Hum Brain Mapp. 2022 Jan 28;43(7):2249–61. doi: 10.1002/hbm.25785 (PMC8996360; doi:10.1002/hbm.25785)
Supplement: Supplementary file 1 — Appendix S1 Supporting Information. [file HBM-43-2249-s001.docx]

Supporting information

Fig. 1 Supp.


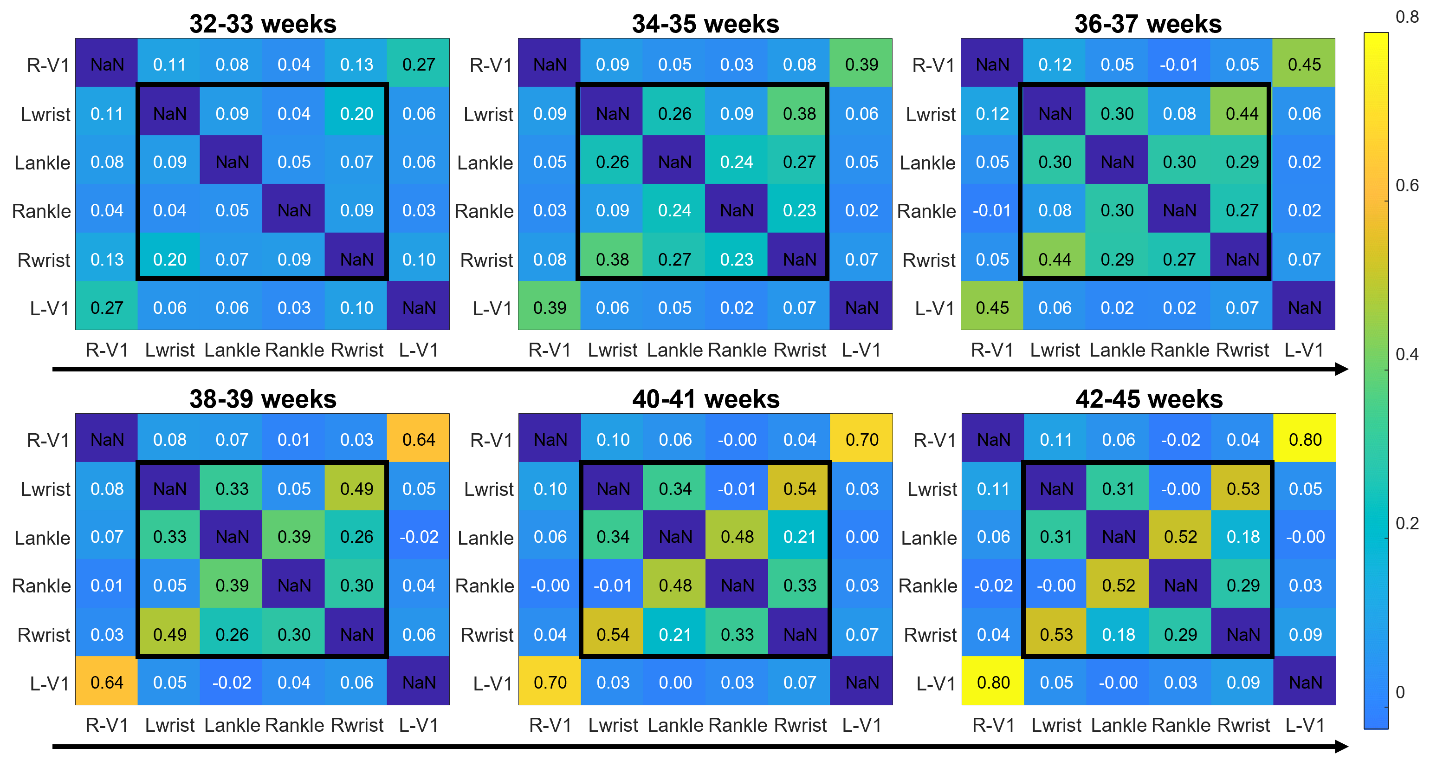


Partial correlation matrices of resting fluctuations between different cortical regions. The inner square highlights regions of the resting-state sensorimotor network while the outer shell shows the connectivity between limbs and a control area (visual cortex). Matrices have been obtained averaging the individual z-transformed partial correlation matrices grouped in different age groups (32≤PMA<34, n=11; 34≤PMA<36, n=28; 36≤PMA<38 n=31; 38≤PMA<40 n=71; 40≤PMA<42, n=152; 42≤PMA<46 n=107).

Table 1 Supp.

| Seed | Average | GA | PMA | sex | motion |
| --- | --- | --- | --- | --- | --- |
| Lwrist | **0.0002** | **0.0002** | **0.0024** | 0.0400 | 0.0059 |
| Lankle | **0.0002** | **0.0002** | **0.0008** | 0.0477 | 0.0063 |
| Rankle | **0.0002** | **0.0002** | **0.0014** | 0.0396 | **0.0012** |
| Rwrist | **0.0002** | **0.0002** | **0.0010** | 0.0424 | **0.0034** |

Summary of p-values obtained from the group seed-based connectivity analysis between each limb region of interest (ROI) and contralateral sensorimotor network. For each ROI, a non-parametric statistical test (using FSL’s built in function Randomise v2.1) investigated the effect of group average (average), gestational age (GA), postmenstrual age at scan (PMA), sex, and number of motion outliers (motion). Bold values indicate the significant cases (α = 0.005 two-tailed). L:left, R: right.

Table 2 Supp.

|  | PMA | pn | PMA*pn | motion |
| --- | --- | --- | --- | --- |
| Lwrist-Rwrist | **5.5 x 10^-7^** | 5.5336 | 4.2082 | **0.0002** |
| Lankle-Rankle | **7.0 x 10^-11^** | 3.0704 | 3.3828 | 0.3052 |
| Lankle-Lwrist | 0.2254 | 3.3662 | 3.9530 | **0.0003** |
| Rankle-Rwrist | **0.0002** | 7.3396 | 5.8711 | 0.0309 |
| Lankle-Rwrist | 0.5323 | 6.1158 | 6.8569 | 0.3113 |
| Rankle-Lwrist | **2.1 x 10^-6^** | 0.6868 | 0.5495 | 2.0844 |
| RV1-Lwrist | 8.6510 | 5.9362 | 6.6018 | **0.0052** |
| RV1-Lankle | 0.8149 | 0.0297 | 0.0259 | 2.5271 |
| RV1-Rankle | 0.0590 | 7.2954 | 7.2241 | 0.9923 |
| RV1-Rwrist | 1.9875 | 0.4263 | 0.5489 | 3.4162 |
| LV1-Lwrist | 3.5058 | 8.2228 | 8.2614 | 7.4273 |
| LV1-Lankle | 0.1515 | 7.3455 | 6.0462 | 4.5086 |
| LV1-Rankle | 2.9944 | 2.2112 | 2.0998 | 7.8826 |
| LV1-Rwrist | 1.6856 | 4.5611 | 4.3860 | 2.0218 |

Summary of p-values obtained from the linear regression analysis between pairs of regions of interest (ROIs). Bonferroni correction was applied to p-values and bold values indicate the significant cases (p*14 <.01). L: left, R: right.

Table 3 Supp.

|  | meanranks | | |  |
| --- | --- | --- | --- | --- |
|  | Preterm | Preterm-at-term | Term | p-value (corrected) |
| Homologs | 181.28 | 363.25 | 413.6707 | **2.9 x 10^-21^** |
| Adjacents | 253.43 | 391.125 | 397.216 | **2.9 x 10^-8^** |
| Distals | 434.56 | 377.3875 | 367.5749 | 0.0712 |
| V1-controls | 1641.98 | 1480.913 | 1489.091 | 0.0176 |

Summary of the results of Kruskal-Wallis H tests of difference in partial correlation coefficients among different cortical area in infants scanned at different age: preterm, preterm-born scanned at term equivalent age, and term. Mean ranks and corrected p-value (p-corrected = p*4) are reported, and in bold are highlighted the significant difference at α =0.01 level.

Table 4 Supp.

|  | GA | PMA Scan 1 | PMA Scan 2 |
| --- | --- | --- | --- |
| 1 | 28.7 | 32.7 | 40.0 |
| 2 | 28.7 | 35.7 | 38.4 |
| 3 | 29.6 | 34.1 | 42.0 |
| 4 | 30.7 | 32.1 | 41.3 |
| 5 | 30.7 | 32.3 | 41.3 |
| 6 | 30.7 | 33.3 | 41.7 |
| 7 | 31.0 | 33.3 | 44.9 |
| 8 | 31.6 | 34.1 | 40.1 |
| 9 | 31.7 | 33.1 | 40.0 |
| 10 | 32.3 | 35.6 | 39.9 |
| 11 | 32.6 | 33.4 | 39.7 |
| 12 | 33.3 | 35.4 | 40.4 |
| 13 | 34.1 | 35.0 | 41.0 |
| 14 | 34.1 | 35.4 | 41.4 |
| 15 | 34.6 | 35.6 | 41.6 |
| 16 | 34.7 | 36.1 | 44.1 |
| 17 | 34.9 | 35.6 | 40.6 |
| 18 | 35.1 | 35.6 | 42.0 |
| 19 | 35.1 | 36.3 | 42.9 |
| 20 | 35.6 | 36.0 | 41.3 |
| 21 | 35.6 | 35.7 | 40.0 |
| 22 | 35.9 | 36.4 | 39.7 |
| 23 | 36.0 | 36.9 | 39.6 |

Demographic data of subjects (n = 23) with repeated measures. Reported gestational age (GA), postmenstrual age at first and second scan (PMA Scan 1, PMA Scan 2).
