# Supplementary material for: Development of functional organization within the sensorimotor network across the perinatal period
Source: Hum Brain Mapp. 2022 Jan 28;43(7):2249–61. doi: 10.1002/hbm.25785 (PMC8996360; doi:10.1002/hbm.25785)
Supplement: Supplementary file 2 — Appendix S2 Supporting Information. [file HBM-43-2249-s002.pdf]

Demographic data of the subjects included in the study

Subject ID	Session ID	Gestational age (weeks)	PMA at scan (weeks)	Sex
CC00112XX05	37001	23	42,42857143	Male
CC00718XX17	235300	24,28571429	41,42857143	Female
CC00518XX15	166600	26,14285714	42,85714286	Male
CC00529AN18	170000	27,42857143	42,28571429	Male
CC00572BN12	161400	27,57142857	44,28571429	Male
CC00661XX10	209600	27,57142857	39	Male
CC00152AN04	49200	28	35	Male
CC00301XX04	113001	28,71428571	40	Female
CC00301XX04	96400	28,71428571	32,71428571	Female
CC00389XX19	133800	28,71428571	38,42857143	Male
CC00489XX20	138800	28,71428571	35,71428571	Male
CC00489XX20	142900	28,71428571	38,42857143	Male
CC00672AN13	214900	28,71428571	41,71428571	Male
CC00672BN13	214800	28,71428571	41,71428571	Female
CC00576XX16	178200	28,85714286	42,14285714	Male
CC00621XX11	195900	29	43,14285714	Male
CC00747XX22	238600	29,42857143	39,42857143	Male
CC00628XX18	181800	29,57142857	34,14285714	Female
CC00628XX18	193500	29,57142857	42	Female
CC00161XX05	53100	29,85714286	36,71428571	Male
CC00578AN18	164900	29,85714286	32,57142857	Male
CC00703XX10	206101	29,85714286	33,71428571	Male
CC00802XX10	1000	29,85714286	32,28571429	Female
CC00855XX14	530	29,85714286	43,57142857	Female
CC00830XX14	30710	30,42857143	39,57142857	Female
CC00124XX09	42302	30,71428571	34,42857143	Female
CC00284AN13	111400	30,71428571	41,28571429	Female
CC00284AN13	90701	30,71428571	32,14285714	Female
CC00284BN13	111500	30,71428571	41,28571429	Male
CC00284BN13	90801	30,71428571	32,28571429	Male
CC00823XX15	15810	30,71428571	33,28571429	Male
CC00823XX15	27810	30,71428571	41,71428571	Male
CC00136AN13	45100	31	33,28571429	Female
CC00136AN13	64201	31	44,85714286	Female
CC00136BN13	64300	31	44,85714286	Female
CC00525XX14	165900	31	40,85714286	Female
CC00293AN14	97401	31,28571429	34	Male
CC00293BN14	97500	31,28571429	34	Male
CC00351XX05	109500	31,28571429	34,57142857	Male
CC00754BN12	223701	31,28571429	38,28571429	Male
CC00617XX15	176500	31,57142857	34,14285714	Male
CC00617XX15	188400	31,57142857	40,14285714	Male
CC00245AN15	94300	31,71428571	43	Male
CC00245BN15	82600	31,71428571	35,14285714	Male
CC00838XX22	21910	31,71428571	33,14285714	Female
CC00838XX22	30610	31,71428571	40	Female
CC00147XX16	48500	32,28571429	35,14285714	Male
CC00361XX07	111700	32,28571429	35,57142857	Male

CC00361XX07	120500	32,28571429	39,85714286	Male
CC00829XX21	17610	32,28571429	33,14285714	Female
CC00154XX06	50700	32,57142857	34	Male
CC00686XX19	198800	32,57142857	33,42857143	Male
CC00686XX19	209100	32,57142857	39,71428571	Male
CC00702AN09	204600	32,57142857	44,14285714	Female
CC00191XX11	79600	32,71428571	40,42857143	Male
CC00889AN24	2220	33,14285714	34,85714286	Male
CC00792XX18	1800	33,28571429	40,42857143	Male
CC00792XX18	244200	33,28571429	35,42857143	Male
CC00788XX22	243000	33,57142857	34,42857143	Male
CC00135AN12	44900	34,14285714	35,14285714	Male
CC00135BN12	44704	34,14285714	35	Female
CC00135BN12	54500	34,14285714	41	Female
CC00569XX17	158300	34,14285714	35,42857143	Male
CC00569XX17	170600	34,14285714	41,42857143	Male
CC00804XX12	900	34,14285714	35	Female
CC00388XX18	118700	34,42857143	41,42857143	Female
CC00907XX16	4230	34,42857143	34,71428571	Male
CC00712XX11	221400	34,57142857	35,57142857	Male
CC00712XX11	232701	34,57142857	41,57142857	Male
CC00764AN14	231800	34,57142857	35,71428571	Female
CC00563XX11	153900	34,71428571	35,28571429	Female
CC00648XX22	191100	34,71428571	36,14285714	Female
CC00648XX22	204400	34,71428571	44,14285714	Female
CC00797XX23	12110	34,85714286	38,85714286	Male
CC00845AN21	26510	34,85714286	35,57142857	Male
CC00845AN21	32010	34,85714286	40,57142857	Male
CC00845BN21	32110	34,85714286	40,57142857	Male
CC00063AN06	15102	35,14285714	35,71428571	Female
CC00407BN11	124100	35,14285714	35,57142857	Male
CC00407BN11	137200	35,14285714	42	Male
CC00492AN15	140900	35,14285714	36,57142857	Female
CC00492BN15	140800	35,14285714	36,57142857	Female
CC00517XX14	145000	35,14285714	36,28571429	Female
CC00517XX14	159200	35,14285714	42,85714286	Female
CC00310XX05	99500	35,42857143	36,85714286	Female
CC00326XX13	118800	35,42857143	42,85714286	Male
CC00271XX08	100400	35,57142857	41,28571429	Female
CC00271XX08	88900	35,57142857	36	Female
CC00385XX15	118500	35,57142857	35,71428571	Male
CC00385XX15	125700	35,57142857	40	Male
CC00570XX10	158900	35,57142857	35,85714286	Female
CC00771XX13	17710	35,57142857	39,71428571	Male
CC00406XX10	122900	35,85714286	36,42857143	Male
CC00406XX10	129200	35,85714286	39,71428571	Male
CC00418AN14	125200	36	36,85714286	Female
CC00418AN14	130200	36	39,57142857	Female
CC00216AN10	73100	36,14285714	36,85714286	Male
CC00216BN10	73200	36,14285714	36,85714286	Male
CC00281AN10	90400	36,14285714	36,85714286	Male

CC00129AN14	43500	36,28571429	36,85714286	Male
CC00129BN14	43701	36,28571429	37	Female
CC00259XX12	85100	36,28571429	36,85714286	Female
CC00186AN14	60900	36,42857143	45,14285714	Female
CC00186BN14	61000	36,42857143	45,14285714	Female
CC00238AN16	80300	36,42857143	36,71428571	Male
CC00238BN16	80400	36,42857143	36,71428571	Male
CC00422XX10	127800	36,42857143	37,14285714	Female
CC00132XX09	44400	36,57142857	37	Male
CC00768XX18	236400	36,57142857	43	Male
CC00237XX15	79300	36,85714286	37,14285714	Female
CC00287BN16	93900	36,85714286	38,71428571	Male
CC00465XX12	137900	36,85714286	37,42857143	Male
CC00627XX17	180600	36,85714286	37	Male
CC00338AN17	107700	37,14285714	37,71428571	Male
CC00338BN17	107600	37,14285714	37,71428571	Male
CC00466AN13	138400	37,14285714	37,85714286	Female
CC00466BN13	138300	37,14285714	37,85714286	Female
CC00109XX10	36901	37,28571429	37,42857143	Female
CC00172AN08	56901	37,28571429	37,85714286	Male
CC00308XX11	98900	37,28571429	37,42857143	Female
CC00647XX21	190801	37,28571429	38,14285714	Female
CC00099AN18	34200	37,42857143	37,71428571	Female
CC00122XX07	42000	37,42857143	38,28571429	Male
CC00150AN02	54800	37,42857143	41	Female
CC00150BN02	49100	37,42857143	37,71428571	Male
CC00337XX16	107000	37,42857143	39,28571429	Male
CC00421AN09	126000	37,42857143	38,14285714	Female
CC00421BN09	126100	37,42857143	38,14285714	Male
CC00412XX08	126301	37,71428571	38	Male
CC00815XX15	4120	37,71428571	42,57142857	Female
CC00170XX06	56100	37,85714286	38,42857143	Male
CC00434AN14	127700	37,85714286	38,85714286	Female
CC00403XX07	124400	38	38,14285714	Male
CC00445XX17	134800	38	38,57142857	Female
CC00520XX09	150201	38	38,28571429	Male
CC00698XX23	220400	38	40,42857143	Male
CC00126XX11	43100	38,14285714	38,28571429	Male
CC00143AN12	47501	38,14285714	38,28571429	Male
CC00143BN12	47600	38,14285714	38,28571429	Female
CC00353XX07	111000	38,14285714	38,42857143	Male
CC00167XX11	55600	38,28571429	43,42857143	Male
CC00587XX19	180300	38,28571429	38,57142857	Female
CC00851XX10	42010	38,28571429	42,71428571	Male
CC00588XX20	183600	38,42857143	41,85714286	Male
CC00064XX07	18303	38,57142857	38,85714286	Male
CC00202XX04	67800	38,57142857	39,28571429	Male
CC00204XX06	69900	38,57142857	38,85714286	Male
CC00299XX20	94900	38,57142857	39	Male
CC00441XX13	132902	38,57142857	41,14285714	Female
CC00810XX10	29010	38,57142857	40,71428571	Male

CC00164XX08	54000	38,71428571	38,85714286	Male
CC00174XX10	57200	38,71428571	41,42857143	Female
CC00355XX09	112600	38,71428571	38,85714286	Male
CC00377XX15	119800	38,71428571	40,71428571	Female
CC00440XX12	132200	38,71428571	41,42857143	Female
CC00547XX20	157300	38,71428571	39	Female
CC00597XX21	190200	38,71428571	39,14285714	Female
CC00708XX15	207500	38,71428571	42	Female
CC00716XX15	222800	38,71428571	40,85714286	Female
CC00782XX16	240100	38,71428571	42,14285714	Female
CC00102XX03	35200	38,85714286	39,14285714	Female
CC00345XX16	109000	38,85714286	39	Female
CC00378XX16	120200	38,85714286	39,57142857	Male
CC00415XX11	127400	38,85714286	39	Female
CC00512XX09	150700	38,85714286	39,14285714	Male
CC00545XX18	170400	38,85714286	43,28571429	Male
CC00744XX19	221001	38,85714286	41,28571429	Female
CC00822XX14	15710	38,85714286	40,57142857	Male
CC00131XX08	44200	39	39,14285714	Male
CC00182XX10	60200	39	41,85714286	Female
CC00207XX09	88700	39	39,14285714	Female
CC00289XX18	119700	39	39,42857143	Female
CC00332XX11	105700	39	41,57142857	Male
CC00335XX14	106300	39	43,14285714	Male
CC00341XX12	108000	39	39,28571429	Male
CC00473XX12	140100	39	39,14285714	Male
CC00482XX13	142000	39	41,14285714	Male
CC00534XX15	155100	39	39,14285714	Female
CC00552XX08	159300	39	39,14285714	Male
CC00740XX15	238400	39	42,85714286	Male
CC00798XX24	245400	39	42	Male
CC00803XX11	2700	39	40,85714286	Female
CC00069XX12	26300	39,14285714	39,57142857	Male
CC00107XX08	36300	39,14285714	41,28571429	Female
CC00114XX07	37100	39,14285714	39,42857143	Male
CC00153XX05	50300	39,14285714	41,57142857	Male
CC00187XX15	61500	39,14285714	41,71428571	Male
CC00188XX16	61800	39,14285714	42,85714286	Male
CC00258XX11	84900	39,14285714	42,85714286	Male
CC00260XX05	85300	39,14285714	39,71428571	Female
CC00344XX15	108600	39,14285714	39,42857143	Male
CC00413XX09	127100	39,14285714	39,28571429	Female
CC00430XX10	131100	39,14285714	44	Male
CC00479XX18	141608	39,14285714	39,42857143	Female
CC00566XX14	164500	39,14285714	42,85714286	Female
CC00595XX19	189900	39,14285714	39,42857143	Female
CC00598XX22	190300	39,14285714	39,28571429	Female
CC00116XX09	38001	39,28571429	39,42857143	Female
CC00157XX09	51900	39,28571429	39,42857143	Male
CC00314XX09	100101	39,28571429	39,42857143	Male
CC00324XX11	111200	39,28571429	39,42857143	Male

CC00376XX14	118400	39,28571429	40,57142857	Female
CC00427XX15	130100	39,28571429	39,85714286	Female
CC00462XX09	134100	39,28571429	40,57142857	Male
CC00649XX23	191201	39,28571429	39,28571429	Male
CC00197XX17	66500	39,42857143	43,14285714	Female
CC00205XX07	70000	39,42857143	39,71428571	Female
CC00362XX08	114500	39,42857143	40,28571429	Female
CC00447XX19	135600	39,42857143	39,71428571	Female
CC00593XX17	189401	39,42857143	40,57142857	Male
CC00669XX18	214300	39,42857143	40,28571429	Male
CC00737XX20	244300	39,42857143	41,42857143	Female
CC00072XX07	27600	39,57142857	40	Female
CC00158XX10	52200	39,57142857	40,28571429	Female
CC00196XX16	66100	39,57142857	42,28571429	Female
CC00232XX10	78300	39,57142857	44,14285714	Female
CC00367XX13	116000	39,57142857	39,85714286	Male
CC00585XX17	178900	39,57142857	39,71428571	Male
CC00592XX16	188800	39,57142857	39,71428571	Male
CC00734XX17	216900	39,57142857	41,85714286	Female
CC00791XX17	27611	39,57142857	43	Female
CC00074XX09	28000	39,71428571	39,85714286	Male
CC00144XX13	47701	39,71428571	40	Male
CC00302XX05	113500	39,71428571	40,85714286	Male
CC00306XX09	98700	39,71428571	40,28571429	Male
CC00443XX15	133900	39,71428571	40,42857143	Male
CC00453XX08	136600	39,71428571	39,71428571	Female
CC00507XX12	148202	39,71428571	40,14285714	Male
CC00568XX16	198900	39,71428571	43,57142857	Male
CC00581XX13	177101	39,71428571	39,85714286	Male
CC00071XX06	27000	39,85714286	40,42857143	Female
CC00100XX01	35000	39,85714286	40	Female
CC00120XX05	41600	39,85714286	40,14285714	Male
CC00247XX17	82801	39,85714286	40,14285714	Female
CC00295XX16	127500	39,85714286	43,42857143	Female
CC00502XX07	146700	39,85714286	40	Male
CC00528XX17	183200	39,85714286	43,71428571	Female
CC00550XX06	157800	39,85714286	40	Female
CC00577XX17	180400	39,85714286	41,71428571	Male
CC00594XX18	189800	39,85714286	40	Male
CC00637XX19	195700	39,85714286	42	Male
CC00115XX08	37801	40	40,28571429	Male
CC00185XX13	60800	40	40,71428571	Female
CC00356XX10	112901	40	40,42857143	Female
CC00371XX09	134700	40	42,57142857	Male
CC00383XX13	121800	40	41,71428571	Female
CC00400XX04	123700	40	40,28571429	Male
CC00411XX07	126200	40	40,57142857	Male
CC00499XX22	145800	40	40,42857143	Male
CC00514XX11	151400	40	40,14285714	Female
CC00555XX11	162400	40	40,28571429	Male
CC00843XX19	4330	40	40,71428571	Male

CC00852XX11	28210	40	43,42857143	Female
CC00168XX12	55700	40,14285714	43,85714286	Male
CC00195XX15	65800	40,14285714	44,28571429	Female
CC00252XX05	84000	40,14285714	43,14285714	Male
CC00265XX10	86901	40,14285714	40,42857143	Male
CC00320XX07	102300	40,14285714	40,28571429	Female
CC00343XX14	108500	40,14285714	42	Male
CC00402XX06	124300	40,14285714	40,42857143	Male
CC00467XX14	139000	40,14285714	41	Male
CC00483XX14	144200	40,14285714	41	Female
CC00509XX14	148800	40,14285714	40,28571429	Male
CC00516XX13	152902	40,14285714	40,57142857	Male
CC00586XX18	179000	40,14285714	40,28571429	Female
CC00719XX18	210600	40,14285714	41,57142857	Male
CC00749XX24	2600	40,14285714	44	Female
CC00879XX22	7430	40,14285714	43,71428571	Female
CC00058XX09	11300	40,28571429	40,57142857	Male
CC00171XX07	56300	40,28571429	40,42857143	Male
CC00189XX17	62301	40,28571429	40,28571429	Male
CC00201XX03	67600	40,28571429	44,14285714	Female
CC00272XX09	117900	40,28571429	42,57142857	Male
CC00321XX08	102700	40,28571429	40,42857143	Male
CC00504XX09	146800	40,28571429	40,57142857	Female
CC00871XX14	38810	40,28571429	41,71428571	Male
CC00065XX08	18600	40,42857143	40,71428571	Male
CC00119XX12	39400	40,42857143	43,71428571	Male
CC00146XX15	48400	40,42857143	40,71428571	Male
CC00165XX09	54600	40,42857143	41,14285714	Female
CC00179XX15	58800	40,42857143	40,57142857	Female
CC00181XX09	60000	40,42857143	40,85714286	Female
CC00267XX12	87700	40,42857143	44,14285714	Male
CC00370XX08	142400	40,42857143	40,85714286	Male
CC00476XX15	141500	40,42857143	44,28571429	Female
CC00542XX15	165800	40,42857143	42,71428571	Female
CC00561XX09	159900	40,42857143	41	Male
CC00564XX12	154100	40,42857143	41,85714286	Male
CC00720XX11	211101	40,42857143	40,57142857	Female
CC00731XX14	214500	40,42857143	44,14285714	Female
CC00765XX15	8210	40,42857143	42,14285714	Male
CC00846XX22	26710	40,42857143	43,28571429	Male
CC00118XX11	39100	40,57142857	40,85714286	Male
CC00178XX14	58600	40,57142857	41	Male
CC00336XX15	106600	40,57142857	44,85714286	Male
CC00428XX16	130400	40,57142857	40,85714286	Male
CC00451XX06	137000	40,57142857	40,85714286	Female
CC00455XX10	137700	40,57142857	42	Female
CC00486XX17	144300	40,57142857	40,71428571	Female
CC00498XX21	144900	40,57142857	42,28571429	Female
CC00505XX10	146900	40,57142857	40,85714286	Male
CC00607XX13	179300	40,57142857	42,42857143	Male
CC00668XX17	220700	40,57142857	41,85714286	Male

CC00840XX16	24910	40,57142857	40,85714286	Male
CC00073XX08	27800	40,71428571	41	Male
CC00110XX03	37000	40,71428571	43,28571429	Male
CC00198XX18	66600	40,71428571	40,85714286	Male
CC00203XX05	69500	40,71428571	40,85714286	Male
CC00206XX08	70100	40,71428571	40,85714286	Female
CC00226XX12	76600	40,71428571	41	Male
CC00233XX11	77800	40,71428571	43,28571429	Female
CC00251XX04	83800	40,71428571	43,57142857	Female
CC00339XX18	107200	40,71428571	41,14285714	Male
CC00408XX12	125500	40,71428571	41	Male
CC00409XX13	125600	40,71428571	41	Male
CC00470XX09	139600	40,71428571	42	Male
CC00511XX08	149000	40,71428571	41	Female
CC00540XX13	164400	40,71428571	41,28571429	Female
CC00650XX07	218007	40,71428571	43,28571429	Female
CC00671XX12	197400	40,71428571	40,85714286	Male
CC00769XX19	4400	40,71428571	41	Male
CC00850XX09	4930	40,71428571	40,85714286	Female
CC00130XX07	44001	40,85714286	43,42857143	Male
CC00134XX11	44600	40,85714286	44,42857143	Male
CC00300XX03	96000	40,85714286	41	Male
CC00366XX12	116300	40,85714286	41,42857143	Male
CC00382XX12	121700	40,85714286	43	Female
CC00417XX13	129000	40,85714286	41,28571429	Male
CC00457XX12	138601	40,85714286	41,42857143	Male
CC00497XX20	144500	40,85714286	41,28571429	Male
CC00554XX10	160200	40,85714286	41,14285714	Male
CC00652XX09	191600	40,85714286	40,85714286	Male
CC00675XX16	219100	40,85714286	42	Female
CC00714XX13	240900	40,85714286	42,71428571	Male
CC00056XX07	10700	41	41,14285714	Male
CC00304XX07	111600	41	41,14285714	Female
CC00357XX11	113900	41	41,71428571	Male
CC00424XX12	129400	41	41,28571429	Female
CC00458XX13	138900	41	41,28571429	Female
CC00500XX05	145900	41	43	Male
CC00527XX16	184400	41	43	Male
CC00057XX08	11002	41,14285714	41,28571429	Female
CC00061XX04	13300	41,14285714	41,42857143	Female
CC00156XX08	51500	41,14285714	41,57142857	Male
CC00159XX11	52600	41,14285714	41,42857143	Female
CC00166XX10	54701	41,14285714	41,42857143	Male
CC00209XX11	70400	41,14285714	42,85714286	Female
CC00217XX11	73700	41,14285714	41,28571429	Male
CC00469XX16	139200	41,14285714	41,42857143	Male
CC00480XX11	141609	41,14285714	44	Female
CC00532XX13	154200	41,14285714	41,42857143	Female
CC00580XX12	173700	41,14285714	41,42857143	Male
CC00589XX21	184000	41,14285714	43,57142857	Female
CC00596XX20	190100	41,14285714	41,57142857	Female

CC00622XX12	185600	41,14285714	41,57142857	Female
CC00818XX18	4020	41,14285714	42,57142857	Female
CC00104XX05	35800	41,28571429	41,71428571	Male
CC00176XX12	57600	41,28571429	43,57142857	Male
CC00184XX12	60501	41,28571429	44,71428571	Female
CC00319XX14	117300	41,28571429	41,71428571	Female
CC00349XX20	110300	41,28571429	42,42857143	Female
CC00381XX11	121600	41,28571429	41,71428571	Male
CC00442XX14	133300	41,28571429	41,57142857	Male
CC00481XX12	141800	41,28571429	41,57142857	Male
CC00535XX16	155600	41,28571429	44,14285714	Female
CC00560XX08	159800	41,28571429	42	Male
CC00639XX21	216800	41,28571429	41,57142857	Female
CC00789XX23	21110	41,28571429	42	Female
CC00113XX06	37200	41,42857143	41,71428571	Female
CC00138XX15	46200	41,42857143	41,57142857	Female
CC00348XX19	110200	41,42857143	41,71428571	Female
CC00461XX08	175100	41,42857143	43,85714286	Female
CC00468XX15	139100	41,42857143	41,85714286	Male
CC00544XX17	169300	41,42857143	41,85714286	Female
CC00117XX10	38200	41,57142857	42,14285714	Male
CC00127XX12	43200	41,57142857	41,85714286	Female
CC00270XX07	88600	41,57142857	41,85714286	Male
CC00446XX18	135200	41,57142857	41,71428571	Female
CC00713XX12	229000	41,57142857	42,85714286	Male
CC00863XX14	34810	41,57142857	43,57142857	Female
CC00180XX08	59500	41,71428571	42,28571429	Male
CC00298XX19	94700	41,71428571	42	Male
CC00342XX13	108300	41,71428571	43,71428571	Female
CC00399XX21	123400	41,71428571	42,85714286	Male
CC00431XX11	131500	41,71428571	43,85714286	Male
CC00444XX16	135101	41,71428571	42,57142857	Female
CC00448XX20	135800	41,71428571	42,14285714	Male
CC00858XX17	32210	41,71428571	43,71428571	Female
CC00193XX13	64400	41,85714286	42	Female
CC00316XX11	101300	41,85714286	44,42857143	Female
CC00352XX06	110700	41,85714286	43,85714286	Male
CC00354XX08	112100	41,85714286	42,42857143	Female
CC00508XX13	148700	41,85714286	42	Male
CC00584XX16	178800	41,85714286	42	Female
CC00685XX18	226100	42	43,28571429	Female
CC00799XX25	23810	42	44	Male
CC00839XX23	23710	42	44,71428571	Male
CC00433XX13	132000	42,14285714	42,42857143	Female
CC00816XX16	40010	42,14285714	42,42857143	Female
CC00656XX13	217601	42,28571429	43	Female
