# Supplementary figures and images for: Development of functional organization within the sensorimotor network across the perinatal period
Source: Hum Brain Mapp. 2022 Jan 28;43(7):2249–61. doi: 10.1002/hbm.25785 (PMC8996360; doi:10.1002/hbm.25785)

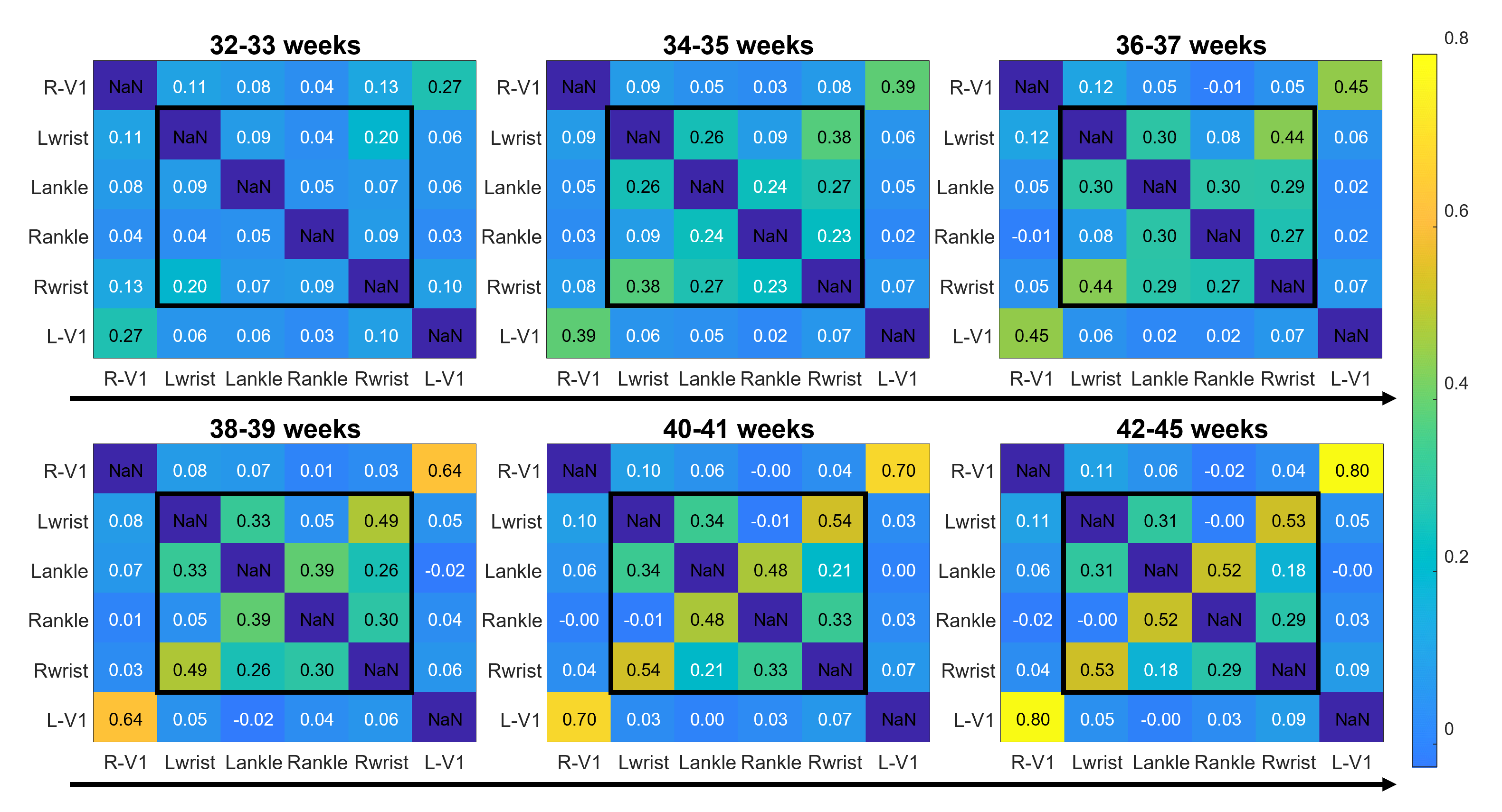

Supplement: Supplementary file 3 — Figure S1 Partial correlation matrices of resting fluctuations between different cortical regions. The inner square highlights regions of the resting‐state sensorimotor network while the outer shell shows the connectivity between limbs and a control area (visual cortex). Matrices have been obtained averaging the individual z‐transformed partial correlation matrices grouped in different age groups (32 ≤ PMA < 34, n = 11; 34 ≤ PMA < 36, n = 28; 36 ≤ PMA < 38, n = 31; 38 ≤ PMA < 40, n = 71; 40 ≤ PMA < 42, n = 152; 42 ≤ PMA < 46, n = 107). [file HBM-43-2249-s003.tif]
